# The association between mechanical ventilation and in-hospital mortality in cardiac intensive care units: A propensity score-matched cohort study

**DOI:** 10.1016/j.clinsp.2025.100728

**Published:** 2025-07-30

**Authors:** XiangTing Tian, Meng Zheng, ShengQing Zhuo, BaoJuan Zheng

**Affiliations:** a Department of Geriatric Cardiovascular, Guangdong Provincial Geriatrics Institute, Guangdong Provincial People’s Hospital, (Guangdong Academy of Medical Sciences), Southern Medical University, Guangzhou City, Guangdong Province, 510080, China; b Guangdong Cardiovascular Institute, Guangdong Provincial People's Hospital (Guangdong Academy of Medical Sciences), Southern Medical University, Guangzhou City, Guangdong Province, China

**Keywords:** Mechanical ventilation, In-hospital mortality, Cardiac intensive care unit, Propensity Score Matching, Critical Care Outcomes

## Abstract

**Objective:**

Mechanical Ventilation (MV) is a common intervention in Cardiac Intensive Care Units (CICUs); however, its effect on in-hospital mortality remains controversial. This study aimed to investigate the association between MV and in-hospital mortality among patients in the CICU using data from the eICU Collaborative Research Database.

**Methods:**

Patients were categorized based on whether they received MV, and propensity score matching was applied to adjust for baseline differences. The primary outcome was in-hospital mortality, which was analyzed using logistic regression and sensitivity analyses.

**Results:**

A total of 12,480 patients in the CICU were included, with 4390 receiving MV and 8090 not receiving MV. In-hospital mortality was notably higher among MV patients (16.0 %) than among those who did not receive MV (4.7 %, *p* < 0.001). After adjusting for confounding factors, MV remained associated with increased mortality, with an odds ratio of 4.16 (95 % CI 3.40‒5.12) in the matched cohort. Sensitivity analyses confirmed these findings, particularly in patients with acute respiratory failure and shock.

**Conclusion:**

These results demonstrate that CICU patients receiving MV have a higher mortality risk, underscoring the need for judicious clinical use of MV.

## Highlights

• Mechanical Ventilation (MV) significantly increases in-hospital mortality in Cardiac Intensive Care Unit (CICU) patients (OR = 4.16).
• Propensity score matching confirmed that MV is independently associated with mortality risk (*p* < 0.001).
• Patients with acute respiratory failure and shock had the highest mortality risk with MV (OR > 4).
• Large-sample analysis based on the eICU database (*n* = 12,480), ensuring reliable results.
• The study supports cautious use of MV in CICU and optimization of respiratory management strategies.

## Introduction

Cardiac Intensive Care Units (CICUs) have evolved significantly in recent decades. Initially focused primarily on acute coronary syndromes, arrhythmias, and heart failure management, modern CICUs now address a broader array of comorbid conditions, including multisystem organ dysfunction and acute non-cardiac illnesses such as sepsis and acute respiratory failure.^1^ The increasing complexity of care within CICUs has led to more frequent utilization of critical interventions such as Mechanical Ventilation (MV) to support patients with life-threatening conditions.^2^

MV is typically employed in patients who experience severe respiratory insufficiency, cardiogenic shock, or cardiac arrest, situations in which the body’s ability to maintain adequate ventilation and oxygenation is impaired. The immediate goal of MV is to provide respiratory support, ensuring that oxygen delivery to vital organs is maintained during critical illness.^3^ Despite its lifesaving role in stabilizing critically ill patients, MV carries a substantial risk of complications, mainly when used for extended periods. These risks include Ventilator-Associated Pneumonia (VAP), Ventilator-Induced Lung Injury (VILI), and adverse hemodynamic effects, all of which can contribute to poorer outcomes and increased mortality.^4^^,^^5^

The annual volume of mechanically ventilated cases in ICUs shows an inverse relationship with in-hospital mortality. A multicenter study revealed that ICUs managing over 400 annual MV cases had 37 % lower mortality compared to those with fewer than 150 cases.^6^^,^^7^ However, conflicting evidence exists, with some studies reporting worse outcomes at higher MV volumes.^8^ When examining Acute Respiratory Failure (ARF), one study differentiated between ARF cases with and without MV. ARF admissions not receiving MV required more resources and showed lower discharge rates than non-ARF cases.^9^ This finding is particularly relevant for CICU patients, who often present with a combination of cardiovascular and non-cardiovascular conditions that may heighten MV-associated risks. In many patients, respiratory failure is frequently secondary to cardiac issues, such as heart failure, ischemia, or arrhythmias.^10^ Therefore, initiating MV in this population requires careful consideration of the potential benefits and risks, as patients may experience immediate oxygenation improvements and a greater likelihood of complications from MV.

Research from non-cardiac intensive care settings has suggested that prolonged MV use is associated with higher rates of morbidity and mortality, especially among patients with multiple comorbidities.^11^^,^^12^ However, there is limited evidence on the impact of MV in CICU-specific populations, where patients often face respiratory issues and cardiovascular instability. Understanding the relationship between MV and mortality in this specialized patient population is essential for guiding clinical decision-making and optimizing outcomes. Therefore, this study aimed to address the knowledge gap by evaluating the association between MV and in-hospital mortality in a large cohort of patients in the CICU. To account for potential confounding factors, propensity score matching was used to ensure that comparisons between MV and no-MV groups were based on similar baseline characteristics.

## Methods

### Study design and data source

This retrospective cohort study utilized data obtained from version 2.0 of the eICU Collaborative Research Database (eICU-CRD), a publicly accessible resource encompassing the clinical information of over 130,000 patients treated in 208 hospitals across the United States between 2014 and 2015.^13^ The database, maintained by the Laboratory for Computational Physiology at the Massachusetts Institute of Technology, offers detailed records of critically ill patients, including those admitted to CICUs. The dataset captures patient demographics, clinical diagnoses coded by the International Classification of Diseases, Ninth Revision (ICD-9), physiological data, and treatments, such as MV and vasopressor administration. All patient identifiers were removed to protect confidentiality, ethical approval was granted by MIT’s institutional review board, and informed consent was waived due to data anonymization.^14^ All clinical studies followed the STROBE statement.

### Patient selection

The study included patients aged 18 years or older who had been admitted to the CICUs with a minimum 24-hour ICU stays. Patients were excluded if critical clinical information, such as APACHE-IV scores, comorbidities, or Oxygen Saturation (SpO_2_) values, were missing, as these were necessary for matching and outcome assessment. For patients with multiple admissions, only the first ICU stay was analyzed to avoid potential bias. Participants were stratified according to MV exposure during their ICU stay. The analysis employed complete-case methodology without data imputation. Missing data analysis revealed exclusion of 47,482 cases (34.07 %) due to incomplete key variables (APACHE-IV score, comorbidities, SpO_2_ values) and 79,411 cases (56.98 %) for other covariates with missing data.

### Data collection

The dataset provided a wide range of variables, including demographics (age, sex, and ethnicity), vital signs (SpO_2_ and blood pressure readings), and APACHE-IV scores, which were used to gauge illness severity upon ICU admission. The study also captured information on clinical interventions, such as vasopressor, sedative, and MV use, along with laboratory values, such as white blood cell count, platelet count, and hemoglobin, in addition to potassium, sodium, Blood Urea Nitrogen (BUN), creatinine, and bicarbonate levels. Comorbid conditions were identified using ICD-9 codes and included coronary artery disease, diabetes, Chronic Obstructive Pulmonary Disease (COPD), sepsis, and acute respiratory failure. Vital signs were recorded during the initial 24 h of CICU admission, and the earliest available laboratory results were included.

### Outcome measures

The primary outcome of interest was in-hospital mortality, which was defined as death before hospital discharge.

### Statistical analysis

Propensity score matching was implemented to address potential confounding from baseline differences between MV and non-MV groups. Propensity scores were generated using logistic regression, accounting for variables such as patient demographics, APACHE IV scores, SpO_2_, blood pressure, and significant comorbidities (sepsis, shock, and respiratory failure). The matching procedure employed a 1:1 nearest-neighbor approach without replacement, using a caliper width of 0.2 times the Standard Deviation (SD) of the propensity score logit^15^ with a caliper value of 0.1, preferentially matching controls with the most similar propensity scores. Matching quality was assessed by examining Standardized Mean Differences (SMDs) of baseline characteristics, with SMDs < 0.1 indicating adequate balance between groups. Although propensity score distributions were not visualized due to sample size limitations, statistical tests verified satisfactory matching quality.

Multivariable logistic regression was used to calculate the Odds Ratios (ORs) and 95 % Confidence Intervals (95 % CIs) for in-hospital mortality, with analyses conducted for both unmatched and matched cohorts. The regression models adjusted for key variables including age, sex, APACHE-IV score, blood pressure, SpO_2_ levels, and major comorbidities (sepsis, shock, and respiratory failure). To assess result robustness, sensitivity analyses were conducted through subgroup stratifications by sex, APACHE-IV score, sedative use status, and presence of acute respiratory failure or shock.

All analyses were performed using R software (version 4.0.1), with statistical significance defined as *p* < 0.05.

## Results

**Fig. 1:**
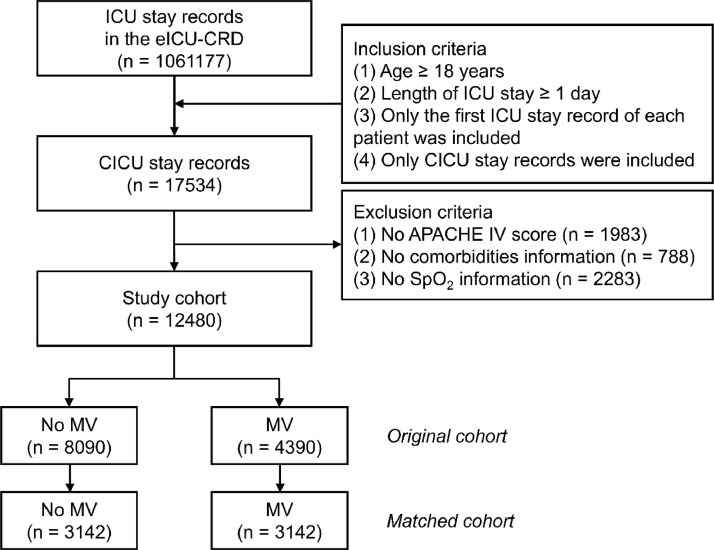
Flow diagram depicting patient selection in the study. A total of 12,480 patients were included after applying the inclusion and exclusion criteria. APACHE, Acute Physiology and Chronic Health Evaluation; CICU, Cardiac Intensive Care Unit; eICU-CRD, eICU Collaborative Research Database; ICU, Intensive Care Unit; MV, Mechanical Ventilation.

**Fig. 2:**
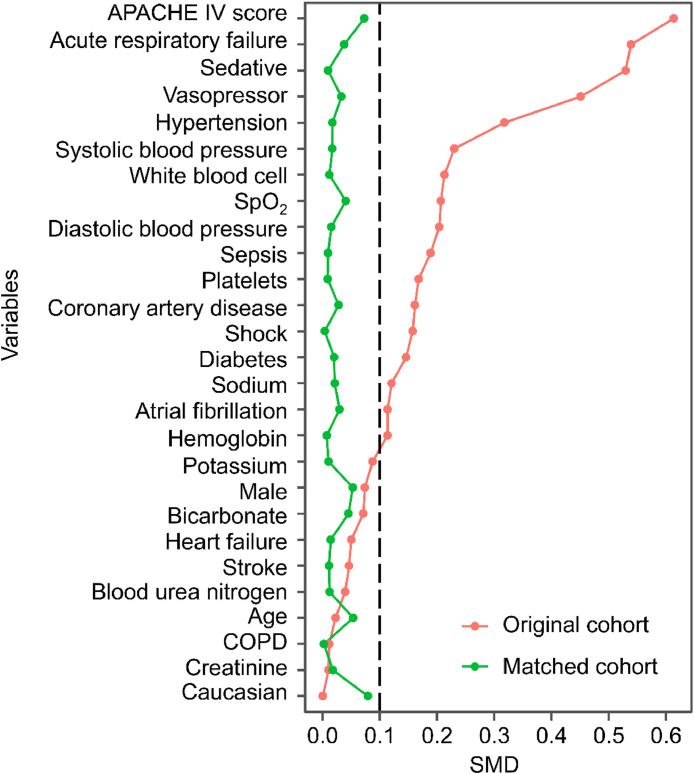
Standardized Mean Differences (SMDs) for clinical variables in the original and matched cohorts. The graph illustrates the balance of variables before and after propensity score matching. APACHE, Acute Physiology and Chronic Health Evaluation; COPD, Chronic Obstructive Pulmonary Disease; SMD, Standardized Mean Difference.

From initially screened cases, 126,887 patients (91.05 %) met exclusion criteria due to ineligibility or missing variables, leaving 12,480 cases with complete data for analysis. After applying the inclusion and exclusion criteria, 12,480 patients were included in the final analysis. Of these, 8090 patients did not receive MV, while 4390 received MV during their CICU stay (Fig. 1). The mean age of the entire cohort was 64.00 ± 14.87 years, and 57.9 % were male. Patients in the MV group had significantly higher APACHE IV scores, indicating greater severity of illness, and were more likely to receive vasopressors and sedatives than those in the no-MV group (Table 1). After propensity score matching, 3142 patients remained in the MV and no-MV groups (Table 2). Before matching, 17 baseline variables had SMDs greater than 0.1, indicating significant differences between the groups. After matching, all SMDs were below 0.1, suggesting that the groups were well-balanced (Fig. 2).

**Table 1:** Baseline characteristics of patients in the original cohort.

Characteristic	No-MV (*n* = 8090)	MV (*n* = 4390)	SMD	p-value
Age, years	64.12 ± 15.17	63.78 ± 14.31	0.023	0.223
Male, n (%)	4578 (56.6)	2644 (60.2)	0.074	<0.001
Caucasian, n (%)	6262 (77.4)	3399 (77.4)	0.001	0.978
SpO_2_, %	96.78 ± 1.97	97.20 ± 2.06	0.207	<0.001
Systolic blood pressure, mmHg	121.25 ± 18.96	117.06 ± 17.42	0.230	<0.001
Diastolic blood pressure, mmHg/mmHg	66.72 ± 12.06	64.37 ± 10.98	0.204	<0.001
APACHE-IV score	48.00 [36.00, 63.00]	60.00 [46.00, 82.00]	0.614	<0.001
Vasopressor, n (%)	1115 (13.8)	1418 (32.3)	0.451	<0.001
Sedative, n (%)	2956 (36.5)	2727 (62.1)	0.529	<0.001
White blood cell count, × 109/L	11.26 ± 6.15	12.63 ± 6.63	0.213	<0.001
Platelet count, × 109/L	215.00 [167.00, 272.00]	200.00 [149.00, 256.00]	0.168	<0.001
Hemoglobin, g/dL	12.19 ± 2.54	11.90 ± 2.43	0.114	<0.001
Potassium, mmoL/L	4.18 ± 0.74	4.25 ± 0.78	0.088	<0.001
Sodium, mmoL/L	136.91 ± 5.33	137.55 ± 5.20	0.121	<0.001
Blood urea nitrogen, mg/dL	20.00 [14.00, 31.84]	19.00 [14.00, 31.00]	0.039	0.290
Creatinine, mg/dL	1.06 [0.81, 1.59]	1.08 [0.82, 1.58]	0.011	0.364
Bicarbonate, mmoL/L	24.31 ± 5.04	23.93 ± 5.39	0.071	<0.001
Coronary artery disease, n (%)	2394 (29.6)	989 (22.5)	0.161	<0.001
Hypertension, n (%)	1660 (20.5)	410 (9.3)	0.318	<0.001
Atrial fibrillation, n (%)	827 (10.2)	309 (7.0)	0.114	<0.001
Heart Failure, n (%)	974 (12.0)	459 (10.5)	0.050	0.008
Stroke, n (%)	188 (2.3)	135 (3.1)	0.046	0.012
Diabetes, n (%)	1068 (13.2)	380 (8.7)	0.146	<0.001
COPD, n (%)	612 (7.6)	319 (7.3)	0.011	0.545
Sepsis, n (%)	1001 (12.4)	844 (19.2)	0.189	<0.001
Shock, n (%)	1389 (17.2)	1031 (23.5)	0.157	<0.001
Acute respiratory failure, n (%)	998 (12.3)	1508 (34.4)	0.539	<0.001

Data are presented as the mean ± standard deviation, median [interquartile range], or number (percentage), as appropriate. APACHE, Acute Physiology and Chronic Health Evaluation; SpO_2_, Oxygen Saturation; COPD, Chronic Obstructive Pulmonary Disease; MV, Mechanical Ventilation; SMD, Standardized Mean Difference.

**Table 2:** Baseline characteristics of patients in the matched cohort.

Characteristic	No-MV (*n* = 3142)	MV (*n* = 3142)	SMD	p-value
Age, years	64.08 ± 15.55	64.86 ± 13.64	0.053	0.034
Male, n (%)	1875 (59.7)	1956 (62.3)	0.053	0.036
Caucasian, n (%)	2450 (78.0)	2550 (81.2)	0.079	0.002
SpO_2_, %	97.06 ± 1.94	96.97 ± 2.14	0.040	0.109
Systolic blood pressure, mmHg	118.06 ± 18.07	117.76 ± 17.69	0.017	0.500
Diastolic blood pressure, mmHg	64.28 ± 11.31	64.45 ± 11.01	0.015	0.542
APACHE-IV score	55.00 [41.00, 71.00]	54.00 [42.00, 72.00]	0.073	0.164
Vasopressor, n (%)	776 (24.7)	821 (26.1)	0.033	0.192
Sedative, n (%)	1669 (53.1)	1654 (52.6)	0.010	0.705
White blood cell count, × 109/L	12.03 ± 7.40	12.11 ± 5.89	0.012	0.636
Platelet count, × 109/L	203.00 [153.00, 260.00]	200.00 [150.00, 256.00]	0.009	0.236
Hemoglobin, g/dL	11.90 ± 2.53	11.92 ± 2.39	0.007	0.773
Potassium, mmoL/L	4.22 ± 0.77	4.23 ± 0.73	0.010	0.678
Sodium, mmoL/L	137.22 ± 5.15	137.33 ± 4.99	0.021	0.400
Blood urea nitrogen, mg/dL	20.00 [14.00, 32.00]	19.00 [14.00, 30.00]	0.012	0.439
Creatinine, mg/dL	1.08 [0.80, 1.60]	1.06 [0.82, 1.54]	0.018	0.301
Bicarbonate, mmoL/L	24.34 ± 5.22	24.10 ± 5.07	0.045	0.072
Coronary artery disease, n (%)	751 (23.9)	789 (25.1)	0.028	0.265
Hypertension, n (%)	331 (10.5)	348 (11.1)	0.017	0.490
Atrial fibrillation, n (%)	232 (7.4)	257 (8.2)	0.030	0.239
Heart Failure, n (%)	348 (11.1)	334 (10.6)	0.014	0.570
Stroke, n (%)	97 (3.1)	91 (2.9)	0.011	0.657
Diabetes, n (%)	272 (8.7)	290 (9.2)	0.020	0.426
COPD, n (%)	224 (7.1)	226 (7.2)	0.002	0.922
Sepsis, n (%)	515 (16.4)	504 (16.0)	0.009	0.707
Shock, n (%)	665 (21.2)	670 (21.3)	0.004	0.877
Acute respiratory failure, n (%)	713 (22.7)	664 (21.1)	0.038	0.135

Data are presented as the mean ± standard deviation, median [interquartile range], or number (percentage), as appropriate. APACHE, Acute Physiology and Chronic Health Evaluation; SpO_2_, Oxygen Saturation; COPD, Chronic Obstructive Pulmonary Disease; MV, Mechanical Ventilation; SMD, Standardized Mean Difference.

**Fig. 3:**
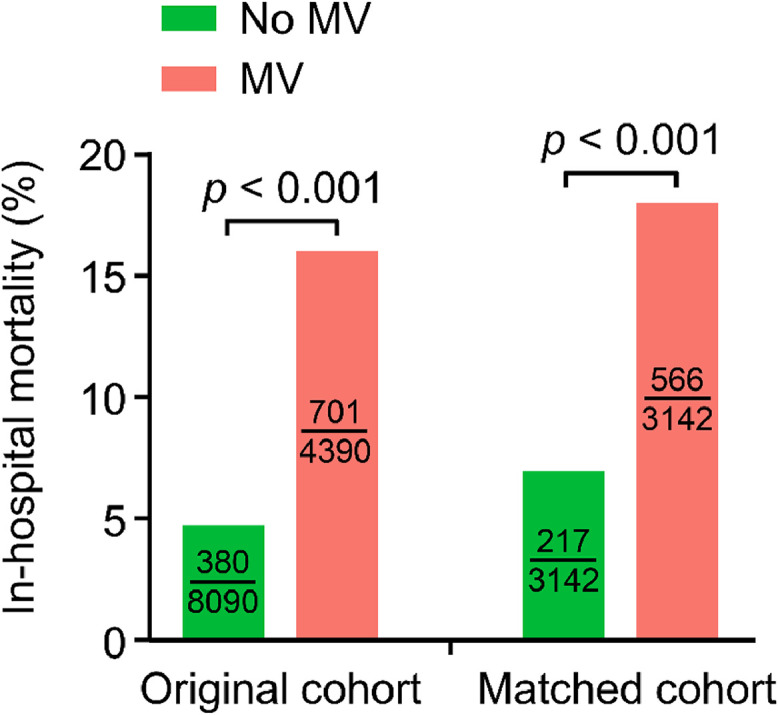
Comparative analysis of in-hospital mortality between MV and no-MV groups. Mortality was defined as the number of deaths among the total number of patients in each group. Statistical significance was assessed using the Chi-Square test. MV, Mechanical Ventilation.

In-hospital mortality was significantly higher in the MV group than in the no-MV group in both the original cohort (16.0 % vs. 4.7 %, *p* < 0.001) and propensity score-matched cohort (16.0 % vs. 4.7 %, *p* < 0.001) (Fig. 3).

**Fig. 4:**
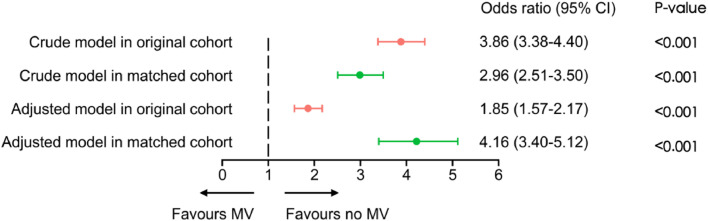
Association between MV and in-hospital mortality in the original and matched cohorts. Logistic regression models were used to calculate Odds Ratios (OR) and Confidence Intervals (CI). MV, Mechanical Ventilation; CI, Confidence Interval.

**Fig. 5:**
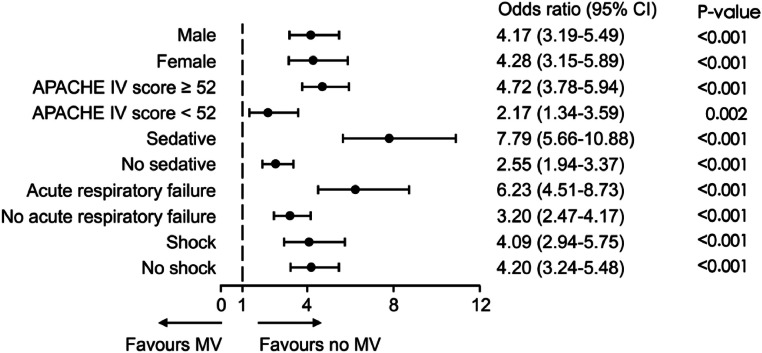
Sensitivity analysis of the association between MV and mortality in a matched cohort. The adjusted model was used to estimate the Odds Ratios (OR) for various subgroups. APACHE, Acute Physiology and Chronic Health Evaluation; MV, Mechanical Ventilation; CI, Confidence Interval.

Logistic regression analysis demonstrated a statistically significant association between MV and in-hospital mortality in both the unmatched and matched cohorts. n the original cohort, MV patients showed an adjusted mortality Odds Ratio (OR) of 1.85 (95 % CI 1.57‒2.17). This association strengthened in the matched cohort (OR = 4.16; 95 % CI 3.40‒5.12) (Fig. 4). Sensitivity analyses confirmed that consistent mortality associations for MV across clinical subgroups: acute respiratory failure (OR = 6.23; 95 % CI 4.51‒8.73) and shock (OR = 4.09; 95 % CI 2.94‒5.75), though formal testing of inter-subgroup differences was not performed (Fig. 5).

## Discussion

The study provides robust evidence that invasive MV is associated with significantly higher mortality risk after confounding factor adjustment. These findings align with previous research showing that complications associated with MV, such as VILI and VAP ‒, are critical factors that may contribute to mortality in critically ill patients.^16^^,^^17^

While MV remains essential for managing respiratory failure and cardiovascular collapse, its application in CICU patients requires careful risk-benefit evaluation. The observed mortality increase may stem from prolonged ventilation's adverse physiological effects, particularly elevated intrathoracic pressure, reduced cardiac output, and hemodynamic instability, all potentially exacerbating underlying cardiovascular conditions.^18^ Therefore, clinicians must cautiously approach using MV, particularly in patients with complex comorbidities.

The relatively high mortality rate observed in the MV group (16.0 %) reflects the severity of the illness in these patients and emphasizes the challenges in managing this high-risk population. Although MV remains essential to stabilize patients, its associated risks demand careful management along with strategies to mitigate complications. Implementing lung-protective ventilation strategies, adopting early weaning protocols, and rigorously preventing ventilator-associated infections could help lower the risks linked to MV and enhance patient outcomes.

Subgroup analyses showed consistently elevated mortality risk with MV in both respiratory failure and shock patients, though interaction effects were not formally tested. These findings underscore the need for individualized care plans, as not all patients respond equally to MV. Certain individuals may be more vulnerable to adverse effects. The current analysis compared effect sizes through stratification without testing interactions, limiting definitive conclusions about differential effects between respiratory failure and shock patients. Future research should aim to identify clinical predictors to guide MV initiation and determine which patient subgroups may benefit most. The study’s findings, derived from eICU-CRD data reflecting specific ventilation strategies and patient characteristics, may not fully apply to other geographic regions or healthcare systems. Extrapolating results may be limited in non-US settings or centers without dedicated CICU infrastructure.^19^ Therefore, clinical implementation should account for local medical resources, treatment protocols, practice patterns, and population characteristics. Regional validation studies are recommended where feasible. Several limitations should be noted. First, the observational design, while reflecting real-world practice, cannot rule out residual confounding or prove causality between MV and mortality. Second, ICD-9-coded comorbidity identification may involve misclassification or underreporting of certain conditions. Third, unmeasured variables ‒ such as specific ventilation parameters (modes, settings) and detailed physiological metrics (blood gases, pulmonary compliance) ‒ could affect outcome interpretation. Despite these constraints, the large sample size and rigorous methodology provide valuable real-world evidence for CICU ventilation management. Future studies should use prospective designs with standardized data collection to evaluate emerging technologies (e.g., non-invasive ventilation, high-flow oxygen) and develop etiology-specific ventilation strategies. Multicenter data integration and advanced electronic health record mining could help overcome current coding limitations. Precision ventilation approaches, tailored to individual patient characteristics ‒ including respiratory failure etiology and cardiovascular comorbidities ‒ need further investigation to strengthen evidence-based respiratory support strategies.

## Conclusions

This propensity score-matched cohort study demonstrated that MV was associated with a significantly increased risk of in-hospital mortality in patients admitted to the CICU. Clinicians should be cautious when deciding to initiate MV, particularly in high-risk patients or those with severe illnesses. Strategies such as lung-protective ventilation and early weaning may help reduce the complications associated with MV. Further research is needed to identify the patients most at risk for poor outcomes and develop targeted interventions to reduce mortality in patients requiring MV.

## Ethics statement

Not applicable.

## Authors’ contribution

XiangTing Tian designed the research study. XiangTing Tian and Meng Zheng performed the research. ShengQing Zhuo and BaoJuan Zheng provided help and advice. Meng Zheng, ShengQing Zhuo and BaoJuan Zheng analyzed the data. XiangTing Tian and Meng Zheng wrote the manuscript. XiangTing Tian reviewed and edited the manuscript. All authors contributed to editorial changes in the manuscript. All authors read and approved the final manuscript.

## Funding

Guangdong Provincial Science and Technology Planning Project (Grant No. 2023B110009).

## Declaration of competing interest

The authors declare no conflicts of interest.

## Data Availability

The datasets used and/or analyzed during the present study are available from the corresponding author on reasonable request.
